# Implications for Electronic Surveys in Inpatient Settings Based on Patient Survey Response Patterns: Cross-Sectional Study

**DOI:** 10.2196/48236

**Published:** 2023-11-01

**Authors:** Megan E Gregory, Lindsey N Sova, Timothy R Huerta, Ann Scheck McAlearney

**Affiliations:** 1 Department of Biomedical Informatics College of Medicine The Ohio State University Columbus, OH United States; 2 The Center for the Advancement of Team Science, Analytics, and Systems Thinking in Health Services and Implementation Science Research (CATALYST) College of Medicine The Ohio State University Columbus, OH United States; 3 Department of Family and Community Medicine College of Medicine The Ohio State University Columbus, OH United States

**Keywords:** surveys, patient satisfaction, patient experience, patient surveys, electronic survey, cross-sectional study, quality of life, mental health, symptoms, data quality, hospitalization

## Abstract

**Background:**

Surveys of hospitalized patients are important for research and learning about unobservable medical issues (eg, mental health, quality of life, and symptoms), but there has been little work examining survey data quality in this population whose capacity to respond to survey items may differ from the general population.

**Objective:**

The aim of this study is to determine what factors drive response rates, survey drop-offs, and missing data in surveys of hospitalized patients.

**Methods:**

Cross-sectional surveys were distributed on an inpatient tablet to patients in a large, midwestern US hospital. Three versions were tested: 1 with 174 items and 2 with 111 items; one 111-item version had missing item reminders that prompted participants when they did not answer items. Response rate, drop-off rate (abandoning survey before completion), and item missingness (skipping items) were examined to investigate data quality. Chi-square tests, Kaplan-Meyer survival curves, and distribution charts were used to compare data quality among survey versions. Response duration was computed for each version.

**Results:**

Overall, 2981 patients responded. Response rate did not differ between the 174- and 111-item versions (81.7% vs 83%, *P*=.53). Drop-off was significantly reduced when the survey was shortened (65.7% vs 20.2% of participants dropped off, *P*<.001). Approximately one-quarter of participants dropped off by item 120, with over half dropping off by item 158. The percentage of participants with missing data decreased substantially when missing item reminders were added (77.2% vs 31.7% of participants, *P*<.001). The mean percentage of items with missing data was reduced in the shorter survey (40.7% vs 20.3% of items missing); with missing item reminders, the percentage of items with missing data was further reduced (20.3% vs 11.7% of items missing). Across versions, for the median participant, each item added 24.6 seconds to a survey’s duration.

**Conclusions:**

Hospitalized patients may have a higher tolerance for longer surveys than the general population, but surveys given to hospitalized patients should have a maximum of 120 items to ensure high rates of completion. Missing item prompts should be used to reduce missing data. Future research should examine generalizability to nonhospitalized individuals.

## Introduction

### Background

Surveys facilitate data collection on unobservable constructs such as symptoms, psychological disorders, and patient experiences. Surveys also enable the collection of important patient information such as health behaviors, and knowledge and understanding of conditions, which may be otherwise difficult to obtain. In the inpatient setting, surveys have been used to assess patient satisfaction [1], patient perceptions of communication [2], and patient willingness to engage in hand hygiene [3]. However, surveys often have low response rates (eg, 6%, 58%, and 32% [1-3]) and high rates of participant drop-off midsurvey [4]. This can lead to low statistical power, increased Type II error [5], and nonresponse bias, defined as “systematic and significant variation between responders and nonresponders” [6]. These biases can result in misleading conclusions [7] and affect the validity of survey findings [8]. High response rates and survey completion rates are therefore essential.

Response rate, defined as the number of individuals who are offered a survey who begin the survey, tends to be low in studies that survey patients. Some research has shown that response rate may be affected by survey length, with longer surveys having a lower response rate compared to shorter surveys [9-11]. Other studies, however, have failed to show this effect [12,13]. Regardless, a high response rate does not guarantee that those who begin the survey provide complete, usable responses.

Drop-off is defined as the number of participants who respond to a survey but do not finish it; that is, the participant abandons the survey before the end. Item missingness is defined as items that a participant does not answer, which may be related to drop-off, but can also include items the participant skipped intentionally or unintentionally. Reasons for drop-off and item missingness are multifaceted; similar to response rate, one possible contributor is survey length and associated response burden. For example, one study found that 10% of participants dropped off a survey almost immediately and completion rates continued to decline for a loss of 2% of participants after every 100 items [4]. Other studies have found similar results, showing that drop-off rates and item missingness are higher for longer surveys compared to shorter surveys [14,15]. Further, poor survey design can also lead to missing data and drop-off [16,17]. At the same time, some aspects of survey design that are common options for web-based surveys can reduce survey drop-off. For instance, prior work has shown that providing motivational reminder statements when participants fail to answer a survey question can reduce the rates of missing data [18]. Additional research suggests that incorporating page breaks leads to higher survey completion rates as compared to using a scrolling design [19].

### Prior Work

Because most of the work on survey response and completion rates has been done outside the hospital setting, little is known about how these findings apply to surveys of hospitalized patients. Hospitalized patients differ from the general population in ways that could either decrease or increase the response rate, drop-off, and item missingness. For instance, a decreased response rate and higher drop-off or item missingness may be expected as many hospitalized patients have low physical or cognitive capacity due to their illness, and this could impact their ability to conduct tasks such as fully completing a survey [20,21]. On the other hand, many hospitalized patients find themselves with free time [22] and in an environment that has limited opportunities for alternative activities that could compete with survey-taking, which could contribute to an increased response rate and lower drop-off or item missingness. While satisfaction surveys of hospitalized patients are a common subject of research, they are generally sent to patients post discharge [23], and thus do not provide insight into surveying patients while in the hospital. Of the few studies that have reported results of surveys conducted in the inpatient environment, patients’ family members or caregivers, rather than the patients themselves, have often been the subject of the survey [24,25]. It is therefore unknown how surveys of the hospitalized patient population can be optimized to increase response rate and reduce drop-off and response burden.

### Research Questions

In this study, we sought to close this gap by examining survey response patterns and drop-off using a sample of over 3500 hospitalized patients to answer the following research questions: (1) Do response rates of hospitalized patients differ as a function of survey length? (2) What is the average survey (i) drop-off rate and (ii) rate of item missingness for hospitalized patients? (3) How does the trajectory of participant drop-off change over the course of a long survey (ie, what is the ideal length of a survey for this population)? (4) What electronic survey design features (eg, page breaks and missing item reminders) are associated with reduced item missingness and drop-off for this population? (5) What is the response burden in this population, in terms of duration (ie, how much time does it take hospitalized patients to complete a survey item)?

## Methods

### Participants

Participants were patients in a large, midwestern US hospital system composed of 6 noncancer hospitals. Data were collected in the context of a randomized controlled trial investigating the impact of an inpatient portal on patient experience [26]. Patients were eligible if they were aged 18 years or older, able to speak or read English, and not involuntarily confined or detained (further details of the sampling strategy are in McAlearney et al [26]).

### Procedure

Patients were provisioned Samsung tablets that provided access to the MyChart Bedside patient portal (Epic Systems). MyChart Bedside is an inpatient portal allowing hospitalized patients to conduct activities such as ordering meals, receiving health education, and taking surveys. Tablets were provisioned no sooner than 6 hours from patients’ admissions, and up to 10 days after admission. Patients were recruited in one of two ways: (1) via an embedded URL on the tablet, or (2) in person by a study team member. Patients were provided with a study overview including the goals of the study, the expectations of participation, and risks and benefits associated with study participation. Patient informed consent was collected via the web with an electronic signature, or via written signature (if patient preference or due to internet connectivity issues).

### Description of the Survey

#### Survey Distribution

The survey was deployed via tablets, through Qualtrics. Up to 50 items appeared on each page of the survey in version A (described further below); versions B and C of the survey (described further below) had approximately 5-10 items per page. Participants were required to hit “next” to continue to proceed through each page of the survey. If participants had only a partial response, Qualtrics recorded their data through the last “next” button they hit. Participants could return to the survey at any time but had up to 2 weeks to complete their responses before Qualtrics closed their survey and stored their incomplete data. No items contained a forced response mechanism (ie, participants could skip any item and still move through the survey). A progress bar showing the percentage of survey completion appeared at the bottom of each page on all versions. At the end of the survey, there was 1 final “next” button, and then participants were shown a screen indicating that the survey was complete.

#### Survey Questions

Analysis of survey items and measures is not the focus of this study. However, the survey included a mixture of item types, including primarily categorical response options (eg, income groups; Likert-type scales), as well as some mark-all-that-apply items, and 3 free response questions. Version A also had one rank order item. See Table 1 for more details on survey topics and items. The survey was comprised of both validated measures (adapted to shorten the scale or revise wording for the patient population as needed) and measures that were internally developed. The purpose of this study was not to assess the reliability or validity of any individual measure in this population. To answer our research questions, items were treated individually, rather than in the context of scales (but grouped into similar concepts as delineated in Table 1, to provide context). As such, we did not compute reliability or assess validity.

**Table 1:** Description of items in each survey version.

	Survey version A	Survey versions B and C
About my care	Items 1-40: (40 items): Access to care, use of care, satisfaction with care, trust in provider, resilience	Items 1-22 (22 items): Access to care, use of care, satisfaction with care, trust in provider, where participants obtained health information
About my health	Items 41-58: (18 items): Health-related self-efficacy and locus of control	Items 23-40 (18 items): Health-related self-efficacy and locus of control
Technology in my life	Items 59-76: (18 items): Access to and use of internet and technology	Items 41-46: (6 items): Access to and use of internet and technology
Using the internet	Items 77-90: (14 items): Willingness to use internet, using internet to search for health information	Items 47-53 (7 items): Willingness to use internet, using internet to search for health information
Seeking health information	Items 91-92: (2 items): Where participants obtained health information	N/A^a^
Using technology to manage my health	Items 93-123: (31 items): Willingness to exchange health information over the internet, and use of patient portals	Items 54-70 (17 items): Willingness to exchange health information over the internet, and use of patient portals
About me	Items 124-174: (51 items): Demographics, health-related quality of life, health literacy and numeracy	Items 71-111: (41 items): Demographics, health-related quality of life, health literacy and numeracy, resilience

^a^N/A: not applicable.

#### Survey Versions

As shown in Table 2, we examined 3 versions of the survey. Version A contained a maximum of 174 items, with a range of 173-174 depending on display logic for 1 item. Survey version B was reduced to a maximum of 111 items (ranging from 100 to 111 items, depending on display logic). Measures were in the same general order for survey versions A and B, but the number of items for each survey topic was reduced (Table 1). In version C, the items remained the same as in version B, but a reminder was added that prompted participants to complete items that were missing when they hit “next” to proceed to a new page. The missing item warning message was the default message developed by Qualtrics; specifically, it read “There are [n] unanswered questions on this page. Would you like to continue?” Participants could choose “continue without answering,” which routed them to the next page of the survey, or “answer the questions,” which took them back to the current page and indicated the incomplete items by highlighting them. If no items on a page were incomplete, the alert did not prompt.

**Table 2:** Survey versions tested in this study^a^.

Survey version	Description of survey version	Purpose of change	Research question (RQ)^b^ tested
A	174 items	N/A^c^	RQ1, RQ2, RQ3, RQ4, and RQ5
B	Reduced to 111 items	To reduce participant burden	RQ1, RQ2, RQ3, RQ4, and RQ5
C	Added prompt to notify participants when they had skipped an item	To reduce missing data	RQ2, RQ3, RQ4, and RQ5

^a^Survey versions were sequential; for example, version C reflected the changes that were made for versions A and B, etc.

^b^RQ (RQ1, RQ2(1), RQ2(2), RQ3, RQ4, and RQ5): research questions.

^c^N/A: not applicable.

#### Survey Implementation

Upon study enrollment, a survey was activated on the tablet within MyChart Bedside and was available throughout the patient’s hospital stay. As part of the randomized, controlled trial, members of the research team visited each enrolled patient up to 3 times to request that they do the survey. In addition, if participants went to the “Getting Started, Getting Involved,” item on the menu tab, a landing page reminded them that they had not completed (or begun) their survey. Participants who began the survey were entered into a monthly raffle for a US $100 gift card.

### Data Analysis

Demographics, including gender, race, age, length of stay, and Charlson Comorbidity Index (CCI), were pulled from the institution’s Information Warehouse (IW) and linked to survey responses. Descriptive statistics for demographics and response rate (defined as the number of participants who were offered the survey and who completed at least 1 survey item), drop-off rate (defined as whether participants responded to the last survey item), and item missingness (defined as whether participants completed every item on the survey) were also computed. Missingness (ie, percentage of items not responded to) within each survey version was also examined via descriptive statistics. Chi-square tests were conducted to compare response rates, drop-off rates, and item missingness by survey version. Kaplan-Meyer Survival curves were computed for each survey version to assess the proportion of respondents who remained in the survey across the items. Distribution charts were developed to map response rates by item.

Duration of the survey response (in minutes) was computed for participants who responded to the last survey item, for each version. The duration was operationalized as time from opening the survey to ending the survey (hitting “submit” or timing out). This was inclusive of any breaks wherein the participant may have been interrupted or closed the survey and returned at a later date or time. This variable was highly negatively skewed, likely due to such breaks. Thus, the medians, IQRs, and the 25th percentiles are interpreted. Analyses were conducted in Stata (version 15; StataCorp) [27].

### Ethical Considerations

The study was conducted in accordance with the Declaration of Helsinki and approved by the institutional review board of The Ohio State University (#2015B0272). Participants provided informed consent and HIPAA (Health Insurance Portability and Accountability Act) authorization.

## Results

### Demographics, Response Rate, and Completion Rates

Overall, 3578 patients were offered the survey, and 2981 completed at least 1 item. Demographics are in Table 3.

The overall response rate was 83.3% (n=2981). Response rate by survey version is shown in Table 4. Response rate did not differ significantly between versions A (174 items) and B (111 items; *χ*^2^_1_=0.39; *P*=.53), addressing research question (RQ) 1**,** and showing that survey length was not associated with response rate in this sample.

The overall percentage of participants who dropped off before the end of the survey was 29.3% (n=873), addressing RQ2(1). The overall percentage of participants who had any missing data (including drop-off but excluding items subject to display logic) was 50.7% (n=1512), addressing RQ2(2). Table 4 provides more details on item missingness by survey version, indicating that the mean percentage of items with missing data ranged from 11.7% (11.4 items; version C) to 40.7% (70.4 items; version A).

Drop-off was substantially reduced from version A (174 items; 65.7%) to version B (111 items; 20.2%). The percentage of participants who had missing data on at least 1 item was high for versions A (621/708, 87.7%) and B (291/377, 77.2%), and decreased substantially for version C (600/1896, 31.7%). Together, these findings suggest that shorter surveys yield less drop-off (*χ*^2^_1_=203.9, *P*<.001 comparing version A to B for responding to the last item), addressing RQ3. In addition, missing item reminders were shown to reduce item missingness (*χ*^2^_1_=273.7, *P*<.001 comparing version B to C for responding to all items), addressing RQ4.

**Figure 1:**
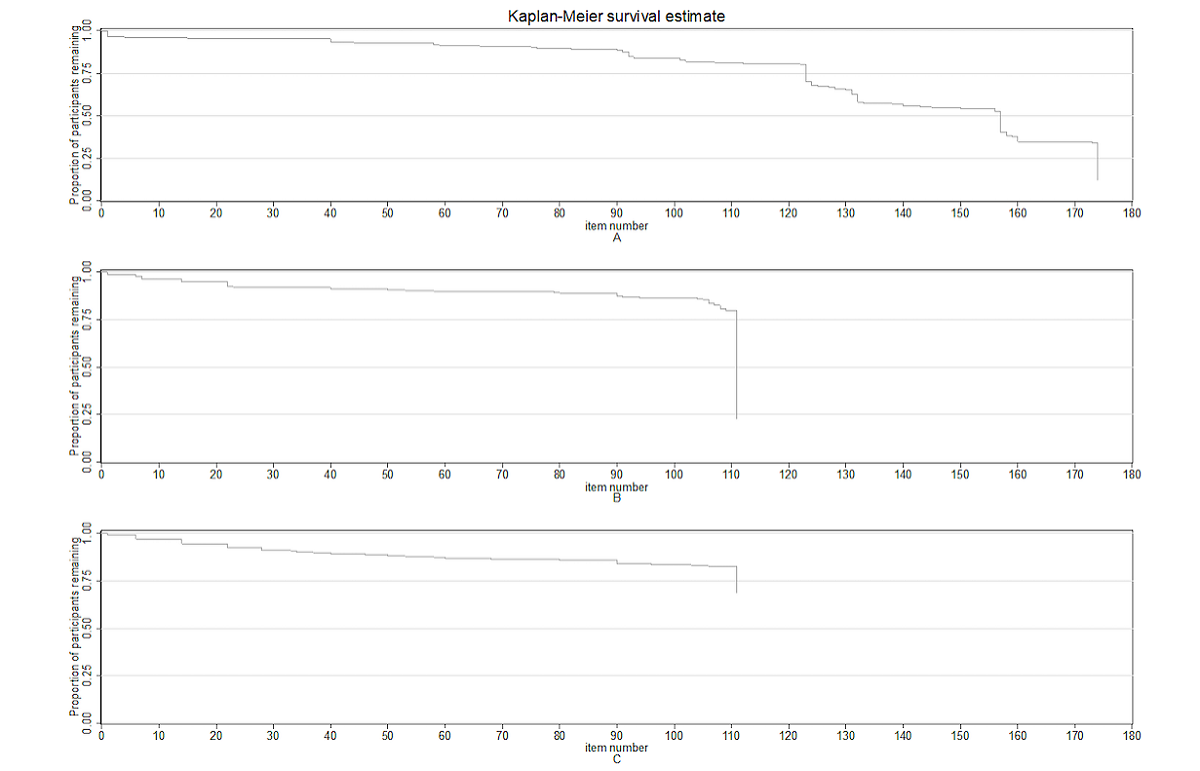
Survival curve showing participants’ drop-off point by survey version. The x-axis indicates the survey item number where participants dropped off the survey, and the y-axis indicates the proportion of participants in each version who still remained in the survey at a given item number. Time of failure=last item responded to. Note that this chart is not reflective of item missingness, that is, participants may have skipped some items throughout the survey before the point of total drop-off.

To investigate RQ3 further, we computed Kaplan-Meier survival curves for each version (Figure 1). The chart indicates that the survival curves for each version trend down slowly, indicating participants slowly drop off as the survey progresses. For version A, the longest survey, which had 174 items, 25% (n=217) of participants had dropped off by approximately item number 120, and more than half of the participants (n=434) had discontinued the survey by item 158.

**Figure 2:**
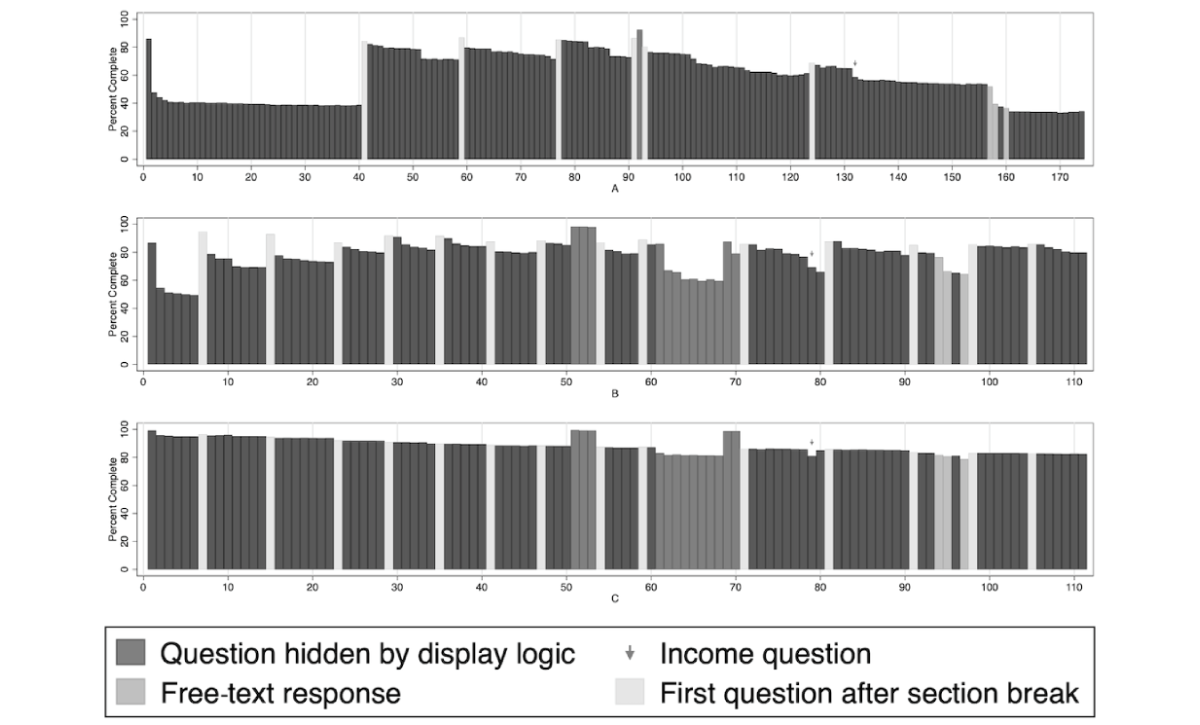
Distribution of item responses, by survey version. For questions with display logic, the denominator is participants who received that question, rather than all participants.

To address RQ4 more fully, we also examined the proportion of responses to each item in each version of the survey in the context of design features such as page breaks (Figure 2). For all versions, there is generally a downward trend of responses to items as the survey progresses. Another common trend is that missing data appears to increase as participants progress within a page, although this effect appears less pronounced in version C (the version where missing item reminders were added). Similarly, for versions A and B, the first items after a page break appear to have higher response rates compared to other items. Relatedly, there are early increases in missing data in versions A and B that recover after page breaks. Related to item type, we noted a higher rate of missing data in the free-response items. We also noted a dip in responses to the question that asks about income, which did not have a “prefer not to respond” option; this effect was present in all survey versions. These trends suggest that both page breaks and reminders about unanswered items are important, particularly in an electronic survey where display cues may not be optimal to prompt participants to scroll for more items.

The proportion of items that are complete versus missing when averaged across respondents differed by survey version. Version A had a large proportion of items that were missing, with a mean of 70.4 (40.7% of items), SD 52.8 (30.5%) items. The number and proportion of missing items decreased substantially in version B, with a mean of 19.9 (20.3% of items), SD 26.7 (27.2%) items, and there was an additional decrease in missing items in version C, with a mean of 11.4 (11.7% of items), SD 24.9 (26.4%) items.

**Table 3:** Demographic and clinical characteristics for patient enrollment admission, by survey version.

Variable			Total (N=2981)		Survey version A (N=708)		Survey version B (N=377)		Survey version C, (N=1896)
Female, n (%)			1784 (59.9)		407 (57.5)		233 (61.8)		1144 (60.3)
**Race, n (%)**									
	Black	527 (17.7)		140 (19.8)		84 (22)		303 (16.0)	
	White	2334 (78.3)		538 (76.0)		280 (74.3)		1516 (80.0)	
	Other	120 (4.0)		30 (4.0)		13 (4.0)		77 (4.0)	
Age (years), median (IQR)			46 (34-58)		48 (35.5-59)		46 (35-57)		46 (33-58)
Charlson Comorbidity Index, median (IQR)			1 (0-3)^a^		2 (0-3)^a^		1 (0-3)		1 (0-2)
Length of stay (days), median (IQR)			5 (3-9)		6 (3-10)		5 (3-9)		5 (3-8)

^a^One patient admission had no associated Charlson Comorbidity Index.

**Table 4:** Response rates, completion rates, and item missingness by survey version^a^.

Result	A: 174 items	B: reduced to 111 items	C: missing item reminders added	Overall
Overall response rate^b^ and sample size for respondents, % (n/n)	81.7 (708/867)	83 (377/454)	84 (1896/2257)	83.3 (2981/3578)
Percent of participants who dropped off^c^, % (n/n)	65.7 (465/708)	20.2 (76/377)	17.5 (332/1896)	29.3 (873/2981)
Percent of participants with item missingness^d^, % (n/n)	87.7 (621/708)	77.2 (291/377)	31.7 (600/1896)	50.7 (1512/2981)
Mean number of items with missing data, mean (SD)	70.4 (52.8)	19.9 (26.7)	11.4 (25.9)	26.5 (42.2)
Mean percentage of items with missing data (%), mean (SD)	40.7 (30.5%)	20.3 (27.21%)	11.7 (26.4%)	19.7 (30.1%)
Mean number of items with complete data, mean (SD)	102.6 (52.8)	78.1 (26.7)	86.6 (25.9)	89.3 (35.2)
Mean percentage of items with complete data (%), mean (SD)	59.3 (30.5%)	79.7 (27.2%)	88.3 (26.4%)	80.4 (30.1%)

^a^Items with display logic were excluded from this analysis.

^b^Defined as response to at least 1 survey item.

^c^Participants who responded to the last survey item, divided by the number of participants who responded to at least 1 survey item.

^d^Participants who responded to every survey item available to them, divided by the number of participants who responded to at least 1 survey item.

### Duration

Duration for version A was the longest, with a median time to completion of 120.9 (IQR 39.8-1333.0) minutes. This corresponds to a median of 41.7 seconds per item. Versions B and C had median durations of 46.2 (IQR 22.7-1162.9), and 42.4 (IQR 24.1-361.0) minutes, respectively. This corresponds to a median of 26.0 seconds per item for version B and 22.8 seconds per item for version C. Overall, across the 3 versions, the median participant took 24.6 seconds per item, indicating that the median participant was able to complete 2-3 items per minute, addressing RQ5.

## Discussion

### Principal Results

The response rate was not related to survey length. However, survey length was associated with completion rate, such that a significantly higher proportion of participants dropped off before the end of the survey in the 174-item version, compared to the 111-item version. The survival curves indicate that an ideal survey length—one in which at least 75% of the participants are retained—may be around 120 items for hospitalized patients. A lower number of items should be used if researchers wish to retain a higher proportion of their sample. Our drop-off rate was higher than that of Hoerger [4], which found an initial drop-off of 10% plus an additional 2% per every 100 items, as we found a drop-off of about 15%-20% in our first 100 items. This difference may be due to the populations included in these studies as Hoerger [4] focused on undergraduate students, who may have been more motivated to complete the survey (eg, as a component of an educational course). Further, it is possible that drop-off was more prevalent in our study due to factors related to our population, such as hospitalized patients possibly feeling too sick to finish the survey. Follow-up work should be done to better investigate this.

Additionally, we found that independent of drop-off, item missingness was prevalent until missing item prompts were added. Before including the prompts, item missingness seemed to occur frequently after the first item on each page, indicating that there may have been a visual issue as participants were not cued to scroll to see additional questions. Regardless of the missing item prompt, the income item was skipped about 20% of the time. This finding is similar to results from prior work showing that about 24% of participants tend to skip income survey questions [28].

Participants took a median of 120.9 minutes to complete the 174-item version of the survey, and 46.2 and 42.4 minutes to complete the different 111-item versions. An important consideration, however, is the context of these findings. Specifically, the duration variable was inclusive of any breaks a participant may have taken while completing their survey—whether for a few moments (eg, due to clinical care), or for hours (eg, if the patient’s tablet needed to be charged at the nursing station). While these durations may be considered more as maximums, they may be realistic in practice for surveys deployed in inpatient environments where interruptions are frequent [29]. These durations are, however, well beyond the recommended optimal survey length of 10-13 minutes [30,31]. Given this, it is surprising that our drop-off was only modest. This suggests that hospitalized patients may have a higher tolerance for longer surveys due to factors such as the lack of other activities in the hospital environment that could compete for their attention. In addition, it is our finding that the median participant was able to answer 2-3 survey questions per minute. Further work should be done to better understand these factors and their implications.

Based on our findings, we present best practices for survey design in Table 5. While these suggestions are developed to guide surveys of hospitalized patient populations, we also indicate the extent to which we believe each best practice can be generalized to other settings. We supplement these suggestions with references that provide additional evidence for each best practice.

**Table 5:** Best practices for survey design.

Best practice recommendation based on this study	Potential generalizability outside inpatient setting	Additional evidence
Limit survey length to approximately 120 items	Hospitalized patients may be a more captive audience than most; needs further study to generalize outside the inpatient setting	Hoerger [4]
Use reminder prompts to alert participants to items they have missed	Likely generalizable	Al Baghal and Lynn [18]; DeRouvray and Couper [32]
Have frequent page breaks such that participant does not need to scroll within a page	Likely generalizable, grounded in research outside the inpatient setting	Manfreda et al [33]; Nosek et al [34]; Peytchev et al [35]; Toepoel et al [36]
Give “prefer not to say” option on income question	Likely generalizable, grounded in research outside inpatient setting	Shah et al [28]
Expect that participants can respond to about 2-3 multiple-choice items per minute	Needs further study to generalize outside the inpatient setting	SurveyMonkey [37]

### Limitations and Future Research

This study has several limitations. First, additional work is needed to examine its generalizability to outpatients and other settings. Second, this study used surveys that were completed on Samsung tablets. It is not clear how these findings generalize to paper surveys, or to surveys taken on a computer or mobile phone. Prior work has established equivalence in responses for tablet, mobile phone, and paper-based surveys [38], but others have found differing response rates [39] and durations [40] between web and mobile surveys. This should be tested in the current context. Further, this study used a survey that was completed by the participants themselves, yet particularly in a hospitalized patient context, family or other caregivers may assist patients with survey completion. Future work should examine differences in response rate, drop-off, missing data, and duration of response in these situations, as it is possible there may be differences. Last, several other factors (eg, demographic and clinical characteristics of patients, patient’s attitude toward surveys, sequence of items in the survey, and participants’ interest in the survey topic) may also impact response rates, survey drop-off, and missing data in the survey, but we were unable to examine these in this study.

### Conclusions

We found that hospitalized patients had a higher tolerance for longer surveys than the general population, with most participants completing at least 120 items. Participants tolerated a median survey duration of 121 minutes for the longest version. In addition, the inclusion of missing item prompts substantially reduced the amount of missing data. Overall, the median participant was able to complete 2-3 items per minute. These findings can be informative for future research when designing surveys for use in the inpatient setting.

## Abbreviations

CCI: Charlson Comorbidity Index
HIPAA: Health Insurance Portability and Accountability Act
IW: information warehouse
RQ: research question

## Data Availability

The data sets generated and analyzed during this study are not publicly available due to patient privacy but are available from the corresponding author upon reasonable request.
